# Comments on dysfunction, activity limitations, participation restriction and contextual factors in South African women with pelvic organ prolapse

**DOI:** 10.4102/sajp.v77i1.1525

**Published:** 2021-01-08

**Authors:** Joyce P. da Silva, Diego Dantas

**Affiliations:** 1 Department of Physiotherapy, Federal University of Pernambuco, Recife, Brazil

We want to highlight some points of the article ‘Dysfunction, activity limitations, participation restriction and contextual factors in South African women with pelvic organ prolapse (POP)’ (Brandt & Janse van Vuuren, 2019).

The article aims to present the biopsychosocial aspects of women with POP in South Africa, using the International Classification of Functioning, Disability and Health (ICF) as a theoretical framework. We congratulate the authors on the relevant theme. However, we need to discuss some points to promote the scientific debate on ICF in this field.

The ICF use has contributed to advancing rehabilitation systems worldwide; ICF codes brought essential data on the consequences of diseases into a biopsychosocial and holistic perspective. Nowadays, there is a call for data production related to the functioning of people in several countries and conditions (Stucki, Rubinelli & Bickenbach 2020).

The ICF (World Health Organization 2001) is a classification system that allows operationalising the expanded concept of health, and universal language is one of the major advantages. Through alphanumeric codes, ICF covers several categories, in different domains of life, which standardise health information and avoid linguistic variations. Therefore, ICF taxonomy is essential in any report involving the ICF.

Regarding this matter, authors in the article do not appropriately use some ICF concepts and do not use any ICF category to represent factors related to POP. The ICF categories are classes and subclasses within a domain of a component, that is, units of classification. For this reason, authors should use the ICF categories to express the issues related to POP.

Throughout the text, the authors used the term ‘dysfunction’ as a synonym for impairments of pelvic floor functions as occurring in this field. While the term ‘dysfunction’ is commonly used when describing pelvic disorders, the term is not included in the ICF and the correct term would be impairments or problems.

According to the ICF, ‘impairment’ is a loss or abnormality in body structure or physiological function (including mental functions); and ‘problem’ is a generic term that can denote impairments, activity limitations and participation restrictions.

Then if the term dysfunction is related to the problems or weakness of the pelvic floor of women with prolapse, the correct way to express this would be impairments or problems in s620 structure of pelvic floor and impairments or problems in b7300 power of isolated muscles and muscle groups.

The authors of the article appear to use the ICF to develop a causative model for POP. However, the ICF does not propose causal models for health conditions but rather is a classification system that allows identifying biopsychosocial factors that contribute, through multiple relationships, to the production of functioning or disability.

The symptoms presented by women with POP should be classified into different ICF components according to their nature. As an example, we could express urinary incontinence secondary to prolapse as being as impairment in the ICF category b620 urinary continence. If prolapses also limit some mobility activities of women, we could in an ICF way say women are showing limitation to activities in the d4 mobility domain. When the prolapse occupies the vaginal cavity, the woman may have difficulty in vaginal sex, exemplifying one participation restriction in d7202 sexual relationships (ICF category).

At the beginning of the discussion, the authors state that *the dysfunctions of pelvic floor muscles reflect impairment component*; however, the ICF has only four components: structures (s), body functions (b), activities and participation (d) and environmental factors (e). According to the ICF taxonomy, the original idea is expressed in three feasible ways. Dysfunctions of pelvic floor muscles are related to impairments in:

- body functions (component),
- b7 Neuromusculoskeletal and movement-related functions (domain or first-level category) and
- b730 Muscle power functions and b750 muscle endurance functions (second-level categories).

The authors propose a framework (Figure 2) to exemplify how the factors and different components of the ICF are interrelated in women with POP. Again, the use of the framework is relevant, but it needs to be updated concerning the correct allocation of factors. We highlight, for example, the item ‘sexual functioning’ allocated as activity limitation in women with POP. However, according to the ICF structure, the content on sexual relations can be understood based on two aspects: as a body function (category b640 – sexual functions) or like participation (category d7702 sexual relationships).

Another emphasis that can be made refers to the ‘poor lifestyle’ which corresponds to a personal factor and not an environmental factor, as stated by the authors. In the same sense, ‘lack physical activity’ cannot be considered an environmental factor, in spite of the three possibilities for classification, depending on the content one wants to express:

- If it refers to the fact that women do not perform the physical activity by their own choice or refer to a sedentary lifestyle, it can be seen as personal factors.
- If it refers to the difficulty of maintaining an adequate level of physical activity despite the woman’s own will, it can be represented as a limitation to the category d570 looking after one’s health.
- If the physical activity to which the authors refer is related to the practice of physical activities for leisure or sports, the category d920 recreation and leisure would be adequate.

Different from what the authors present, emotional factors and issues related to sleep or energy, according to the ICF, can be represented by specific categories, such as b134 sleep functions, from the domain b1 mental functions, related to the component body functions (b) and not activity and participation as mentioned.

As shown in the example above, identifying ICF categories to express common items in the patient’s evaluation is possible. In this sense, when linking health information with ICF categories, it is strongly recommended that the use of widely reported and previously published methods (Cieza et al. 2002, 2005, 2016) in studies for this purpose are considered in order to generate adequate data, information and comparable data on patients as well as different clinical contexts.

We appreciate the opportunity to explain our comments on the correct use of the ICF and contribute to the familiarisation and better understanding of the classification system and its potential use. We recommend that authors who aim to describe factors related to health contexts use the endorsed methodologies, observing the ICF taxonomy and structure to avoid the risk of errors and making the popularisation of the ICF amongst professionals and researchers more challenging.
